# Once-weekly hemodialysis combined with low-protein and low-salt dietary treatment as a favorable therapeutic modality for selected patients with end-stage renal failure: a prospective observational study in Japanese patients

**DOI:** 10.1186/s12882-018-0941-2

**Published:** 2018-06-28

**Authors:** Toshiyuki Nakao, Yoshie Kanazawa, Toshimasa Takahashi

**Affiliations:** 1 Department of Clinical Research, Organization for Kidney and Metabolic Disease Treatment, 1-32-1, Okusawa, Setagaya ward, Tokyo, 158-0083 Japan; 2 Department of Human Nutrition, Tokyo Kaseigakuin University, 22, sanbanchou, Chiyoda ward, Tokyo, 102-8341 Japan; 3 Bousei Shinjuku- minamiguchi Clinic, 2-9-2 Yoyogi, Shibuya, Tokyo, 151-0053 Japan

**Keywords:** Once-weekly hemodialysis, Infrequent dialysis, Low protein diet, End-stage renal failure, Dialysis adequacy

## Abstract

**Background:**

For patients with end-stage renal failure (ESFR), thrice-weekly hemodialysis is a standard care. Once-weekly hemodialysis combined with low-protein and low-salt dietary treatment (OWHD-DT) have been rarely studied. Therefore, here, we describe our experience on OWHD-DT, and assess its long-term effectiveness.

**Methods:**

We instituted OWHD-DT therapy in 112 highly motivated patients with creatinine clearance below 5.0 mL/min. They received once-weekly hemodialysis on a diet of 0.6 g/kg/day of protein adjusted for sufficient energy intake, and less than 6 g/day of salt intake. Serial changes in their clinical, biochemical and nutritional parameters were prospectively observed, and the weekly time spent for hospital visits as well as their monthly medical expenses were compared with 30 age, sex- and disease-matched thrice-weekly hemodialysis patients.

**Results:**

The duration of successfully continued OWHD-DT therapy was more than 4 years in 11.6% of patients, 3 years in 16.1%, 2 years in 24.1% and 1 year in 51.8%. Time required per week for hospital attendance was 66.7% shorter and monthly medical expenses were 50.5% lower in the OWHD-DT group than in the thrice-weekly hemodialysis group (both *p* < 0.001). Patient survival rates in the OWHD-DT group were better than those in the Japan Registry (*p* < 0.001). Serum urea nitrogen significantly decreased; hemoglobin significantly increased; and albumin and body mass index were not significantly different from baseline values. In the OWHD-DT patients, serum albumin at 1 and 2 years after initiation of therapy was significantly higher compared with prevalent thrice-weekly hemodialysis patients. Furthermore, residual urine output was significantly higher in the OWHD-DT patients than in those receiving thrice-weekly hemodialysis (*p* < 0.05). Interdialytic weight gain over the course of the entire week between treatments in patients on OWHD-DT were 0.9 ± 1.0, 2.0 ± 1.3, 1.9 ± 1.2, 1.9 ± 1.5 and 1.8 ± 1.0 kg at 1, 6, 12, 18 and 24 months, respectively, though the weekly weight gain for thrice-weekly hemodialysis group (summed over all 3 treatments) was 8.6 ± 0.63 kg, *p* < 0.001.

**Conclusions:**

OWHD-DT may be a favorable therapeutic modality for selected highly motivated patients with ESRF. However, this treatment cannot be seen as a general maintenance strategy.

**Trial registration:**

UMIN000027555, May 30, 2017 (retrospectively registered).

## Background

Hemodialysis (HD) three times per week is the conventional standard method to treat patients with end-stage renal failure (ESRF) in whom uremia can no longer be managed conservatively. Argument on dialysis adequacy has always been based on thrice-weekly HD for the last 30 years, [1–3], and less frequent dialysis has rarely been the subject of study. Recently, however, twice-weekly HD has been proposed as a new paradigm for initiation of HD in ESRF patients with significant residual kidney function [4–6]. Clinical guidelines approve this approach in certain settings [7, 8]. Irrespective of dialysis frequency, standard recommended dietary protein intake is 1.0–1.2 g/kg/day [9, 10], which is higher than that recommended for healthy populations [11].

A low-protein diet has long been used for reducing the retention of nitrogenous toxic metabolites in chronic renal failure [12–15]. Dietary treatment (DT) consisting of low-protein and low-salt intake can forestall the development of uremic symptoms, and has played a central role in the conservative management of ESRF for decades [16–18]. Some patients show good adherence to DT, and thus can successfully postpone the initiation of dialysis [19–21]. Because these patients have put significant effort into their own health, the sudden transition from conservative therapy with a low-protein diet to conventional thrice-weekly HD with a usual-protein diet (C-HD) can be psychologically unacceptable, despite the deterioration of their renal function to critical levels. In these cases, a staged transition by which HD is given at a low frequency might be considered.

One session of HD for ESRF patients can remove excess body fluids and decrease accumulated metabolites to near normal levels. However, during the interval between HD sessions, fluids and toxic metabolites accumulate in the body through the ingestion of foods and drinks. Under these circumstances, DT with a low-protein and low-salt diet can slow the accumulation of fluids and toxic metabolites, thus increasing the necessary intervals between HD. It seems reasonable, therefore, that a combined treatment of infrequent HD and DT would be worth trying as an alternative to C-HD.

To date, however, only a few studies on the combined treatment of infrequent HD and DT, have been reported [22–27]. Therefore, in this study, we describe our experiences with a combined therapy consisting of once-weekly hemodialysis and DT with a low-protein and a low-salt intake (OWHD-DT). We then assessed the long-term effectiveness of this alternative therapy.

## Methods

### Patients

Between 1992 and 2014, 112 consecutive patients treated with OWHD-DT at Tokyo Medical University Hospital and Bousei Shinjuku-minamiguchi Clinic, and followed until the end of 2016, were enrolled in this study.

Criteria for patient selection was as follows: 1) creatinine clearance below 5.0 mL/min or serum creatinine over 8.0 mg/dL, 2) clinical emergence of uremic gastrointestinal symptoms or other uremic symptoms on conservative treatment, and 3) patient willingness and high motivation to choose the therapeutic modality, OWHD-DT, with informed consents. Patients who met all three criteria were instituted to OWHD-DT. They were changed to C-HD if either they did not remain motivated to adhere to the required DT, or if their residual urine output decreased to ≦500 mL/day with concomitant concern for the emergence of uremic symptoms necessitating an increased rate of dialysis.

On October, 2013, 30 outpatient on C-HD at Bousei Shinjuku-minamiguchi Clinic were recruited, in order to compare their overall monthly medical expenses, the weekly time required for medical treatment including both dialysis and hospital attendance, daily urine output, the weekly interdialytic weight gain (IDW) and the dietary salt and protein intake, with the patients on OWHD-DT. In comparison of IDW, the average over 4 weeks of IDW was examined. The weekly IDW was the sum of weight gains for all 3 treatments per week for patients on C-HD, and was the weight gain for the entire week between once-weekly treatments for patients on OWHD-DT. Salt intake was calculated as (IDW × serum sodium concentration + urinary sodium excretion)/17, and protein intake was calculated from patients’ diet dairies.

Written informed consent was obtained from all patients, and the studies were conducted in accordance with the principles set out in the Declaration of Helsinki regarding clinical trials with human subjects. The study was approved by the ethics committee of Tokyo Medical University.

### Hemodialysis

HD was conducted every 7 days, using high-flux polysulfone membrane dialyzers, at a blood flow rate of 200–250 mL/min and a dialysate flow rate of 500 mL/min. The length of each dialysis session was 3.5 to 5 h. The fluid removal rate varied according to the volume accumulated in the patient, and was kept constant from the beginning to the end of the HD session.

### Dietary treatment

The patients were advised to follow a low-protein, low-salt diet with sufficient energy intake and received nutritional counselling by a renal dietician once or twice a month. The prescribed diet consisted of daily intake of 0.6 g/kg standardized body weight of protein, with approximately 65% of the dietary protein of high biological value (i.e., from animal sources). Prescribed salt intake was less than 6 g/day. A 6 g portion of salt contains 2.4 g of sodium. Energy intake consisting primarily of carbohydrates and fats was individually prescribed for each patient, in equilibrium with estimated energy expenditure as determined by their sex, age and physical activity. The average prescribed daily energy intake was 32 ± 2 kcal/kg.

A skilled dietitian trained the patients to follow the diet, including how to use specially manufactured protein-reduced energy-equivalent rice, noodles, and bread products to make up for energy lost through decreased protein intake. Protein-reduced rice was manufactured by treating ordinary rice by enzymes that degrade proteins to the level of 1/25th, followed steaming, and packing in 200 g plastic container. Protein-reduced bread and pasta were prepared from corn starch, gum, yeast and sugar. The patients on OWHD-DT purchased those specifically manufactured foods by themselves. Patients not on the protocol did not obtain similar diets. If a patient not on the protocol wanted to consume this diet, he/her can purchase the foods through http://www.healthynetwork.co.jp

Fluid intake was interviewed by nurses at the time of every dialysis treatment. Strict restriction of water intake was not instructed; instead salt restriction was strictly instructed and repeatedly consulted, since intake of salt directly results in ingestion of water in ESRF patients. A typical diet for a single day for an OWHD-DT patient is shown in Table 1. Adherence to the low-protein/low-salt diet was evaluated monthly by a skilled dietitian, YK, based on the patients’ diet diary, interview with the patients and 24-h urine collection.

**Table 1:** A typical diet for a single day for an OWHD-DT patient

			g
Breakfast			
	Low protein bread	low protein bread	100
		strawberry jam	15
	Fried egg	egg	50
		oil	3
		salt	0.5
		pepper	0.02
		cabbage	30
		tomato	20
		mayonnaise	5
	Fruit	orange	50
	Beverage	nutritional formula ^47)^	125
Lunch			
	Spaghetti	low protein spaghetti	100
		mushroom	20
		onion	30
		garlic	3
		olive oil	8
		soy sauce	6
		salt	1
		pepper	0.02
	Grilled beef	tenderloin	55
		salt	0.3
		oil	3
		tomato	30
	Black tee	black tee	130
		sugar	6
	Jelly	jelly	100
Dinner			
	Low protein rice	low protein rice	200
	Grilled salmon	salmon	50
		salt	0.5
		low protein bread flour	6
		oil	10
		lettuce	15
		cucumber	30
		lemon	5
		mayonnaise	5
	Salad	carrot	50
		olive oil	3
		salt	0.3
	Dessert	sherbet	80
		strawberry	30

Total energy 2067 kcal, protein 34.8 g, fat 64 g, carbohydrate 344.1 g potassium 1290 mg, phosphorus 540 mg, salt 5.5 g, amino acid score 100

*OWHD-DT*, Once-weekly hemodialysis combined with dietary treatment

### Laboratory measurements

Blood samples and blood pressure were obtained at the start of HD. 24-h urine collections were examined monthly. Serum albumin was measured by the BCG method. A chest radiograph was obtained just after HD, and cardiothoracic ratio was measured. Body mass index (BMI) was calculated as body weight (kg) divided by height squared (m^2^). The calculation of BMI was based on body weight just after HD.

### Statistical analyses

Data are expressed as means±SD. Testing for statistical significance was conducted using the t-test for parametric variables and the Mann-Whitney U test for nonparametric variables. A *p*-value of < 0.05 was considered to be statistically significant. A Kaplan-Meier curve was drawn to present the survival of the patients. SPSS statistical software (version 21; IBM Corporation, Chicago, IL) was used for all calculations.

## Results

### Patient characteristics

Median age of the patients was 63 (interquartile range: 53–69) years old at the start of OWHD-DT. Diseases leading to renal failure, vascular access and prescribed medications at the initiation of OWHD-DT were shown in Table 2. A total of 97.3% of the patients began OWHD-DT at an outpatient clinic and the other 2.7% on admission to the hospital. Creatinine clearance of the studied patients at the initiation of HD was 3.8 ± 0.8 mL/min, and urea clearance 2.3 ± 0.7 ml/min. Mean single pool Kt/V (spKt/V) per a HD session was 1.34 ± 0.13.

**Table 2:** Patient characteristics at the initiation of OWHD-DT

Characteristic	Total *n* = 112
Age (yr)	63 (53–69)
Men, *n* (%)	80 (71.4)
Diseases, *n* (%)	
Chronic glomerulonephritis	38 (33.9)
Diabetic nephropathy	38 (33.9)
Nephroscrelosis	22 (19.6)
Polycystic kidney	9 (8.0)
Urological disease	3 (2.7)
Chronic interstitial nephritis	2 (1.8)
Pretreatment, *n* (%)	
Conservative therapy	97 (86.6)
Twice-weekly hemodialysis	14 (12.5)
Thrice-weekly hemodialysis	1 (0.9)
Vascular access, A-V fistula, *n* (%)	112 (100)
Medications, *n* (%)	
Duretics	107 (95.5)
Erythropoesis stimulating agent	111 (99.1)
Antihypertensives	87 (77.7)
Phosphate binders	84 (75.0)
Activated vitamin D	89 (79.5)
Alkalizing agents	62 (55.3)
K+ ion-exchange resin	15 (13.3)

*OWHD-DT*, Once-weekly hemodialysis combined with dietary treatment

### Outcomes

The patients on OWHD-DT were changed to twice-weekly or thrice-weekly HD, when they became unable to adhere the DT or when their residual urine output decreased to ≦500 mL/day with a concomitant fear of the emergence of uremic symptoms. Continuation and outcomes are shown in Table 3, and the longest was 11.5 years. 38 out of 112 (33.9%) patients quit the protocol because they did not remain motivated to adhere to the diet. Especially for the 26 patients who quit the protocol within 6 months, the most reason (96.2%) of discontinuation was poor adherence to the diet, mainly because of palatability for the diet and frequent eating out. On the other hand, in all 39 patients on OWHD-DT over 18 months or longer, adherence to the diet was excellent, and their reasons of discontinuing the protocol were reduction of residual urine outputs.

**Table 3:** Continuation and outcomes

Time (months)	Number of patients on the OWHD-DT *n*, (%)	Outcomes
0	112 (100)	
6	86 (76.7)	26 transferred to C-HD
12	58 (51.8)	27transferred to C-HD, 1 died
18	39 (34.8)	18 transferred to C-HD, 1 transplanted
24	27 (24.1)	11 transferred to C-HD, 1 died
30	20 (17.8)	7 transferred to C-HD
36	18 (16.1)	2 transferred to C-HD
42	15 (13.4)	3 transferred to C-HD
48	13 (11.6)	2 transferred to C-HD
54	11 (9.8)	2 transferred to C-HD
> 60	10 (8.9)	1 transferred to C-HD

*OWHD-DT*, Once-weekly hemodialysis combined with dietary treatment; *C-HD*, Conventional twice- or thrice- weekly dialysis

**Fig. 1:**
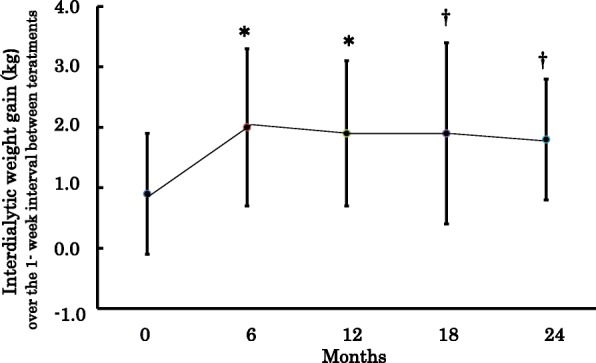
Interdialytic weight gain (IDW) for the entire week between treatments in the patients who remained on once-weekly hemodialysis and diet therapy (OWHD-DT). The IDW value of each patient is the mean of 4 values for the entire week between treatments at follow-up time points. ✻*p* < 0.001, † *p* < 0.05 vs. baseline

Serial changes in biochemical and clinical parameters after beginning of OWHD-DT are shown in Table 4. Interdialytic weight gain (IDW) were 0.9 ± 1.0, 2.0 ± 1.3, 1.9 ± 1.2, 1.9 ± 1.5 and 1.8 ± 1.0 kg at the first month, 6, 12, 18 and 24 months, respectively (Fig. 1). The values of IDW are for the entire week between treatments. The IDW value of each patient is the mean of 4 values for the entire week between treatments at follow-up time points. On the other hand, the weekly IDW for CH-D group (summed over all 3 treatments) was 8.6 ± 0.63 kg, *p* < 0.001.

**Table 4:** Biochemical and clinical parameters in patients on OWHD-DT at follow-up time points during 24 months of the study

Duration (months)	0 M	6 M	12 M	18 M	24 M
Number of the patients	112	86	58	39	27
Urea nitrogen (mg/dl)	98.5 ± 30.0	83.9 ± 17.0§	85.4 ± 23.2#	85.7 ± 22.1#	77.2 ± 15.8†
Creatinine (mg/dl)	11.7 ± 4.0	12.2 ± 4.1	12.5 ± 3.8#	12.8 ± 4.3†	12.6 ± 4.4#
β2 microglobulin (mg/L)	19.5 ± 5. 6	23.1 ± 7.7†	23.3 ± 7.2†	23.5 ± 7.3	24.4 ± 7.5
Sodium (mEq/l)	136.6 ± 5.6	138.6 ± 3.8†	138.5 ± 3.6†	138.9 ± 4.2†	139.2 ± 4.6
Potasium (mEq/l	4.3 ± 0.8	4.6 ± 0.8#	4.7 ± 0.9§	4.6 ± 0.7 #	4.8 ± 0.8†
Calsium (mg/dl)	8.0 ± 1.2	8.9 ± 1.2§	8.9 ± 1.2†	8.5 ± 0.4§	8.7 ± 0.4#
Posphate (mg/dl)	6.4 ± 1.9	5.8 ± 1.7	6.0 ± 1.5	5.7 ± 1.3	5.8 ± 1.4
Bicarbonate (mEq/l)	18.7 ± 4.7	21.8 ± 2.3	22.3 ± 3.3	21.9 ± 1.9	22.0 ± 2.9
Hemoglobin (g/dl)	8.7 ± 1.2	10.3 ± 1.5§	10.0 ± 1.6#	10.6 ± 0.8†	10.6 ± 1.2†
Albumin (g/dl)	3.9 ± 0.5	4.1 ± 0.4	4.1 ± 0.4	4.0 ± 0.3	4.0 ± 0.3
Transferin (mg/dl)	204.1 ± 45.4	211.8 ± 45.6	214.1 ± 37.5	212.6 ± 42.7	209.3 ± 34.7
BMI (kg/m2)	20.9 ± 2.6	21.1 ± 2.6	21.1 ± 2.9	20.6 ± 2.7	20.7 ± 2.6
iPTH (pg/ml)	263.1 ± 204.4	151.1 ± 141.4§	129.9 ± 79.5#	132.5 ± 68.7#	142.1 ± 146.4#
CRP (mg/L)	1.27 ± 1.18	1.11 ± 0.98	1.52 ± 1.63	1.45 ± 1.63	1.05 ± 1.21
Systolic blood pressure (mmHg)	154.0 ± 19.9	156.3 ± 20.4	153.7 ± 16.9	154.0 ± 20.1	150.5 ± 17.6
Diastolic blood pressure (mmHg)	81.8 ± 12.2	84.1 ± 12.4	83.3 ± 10.8	83.1 ± 11.7	81.8 ± 12.1
CTR (%)	49.6 ± 5.9	49.1 ± 4.4	49.5 ± 5.3	48.5 ± 4.8	48.3 ± 3.7

*OWHD-DT*, Once-weekly hemodialysis combined with dietary treatment; *BMI*, Body mass index; *iPTH*, Intact parathyroid hormone; *CRP*, C-reactive protein; *CTR*, cardiothrasic ratio

§*p* < 0.001; #*p* < 0.01; †*p* < 0.05 vs. baseline

Actual daily salt intake was 6.3 ± 1.5 g in the patients on OWHD-DT at the first month vs. 13.7 ± 2.7 g in C-HD group, p < 0.001. Actual daily protein intake was 0.65 ± 0.06 g/kg in the patients on OWHD-DT at the first month vs. 1.04 ± 0.12 g for C-HD group, *p* < 0.001.

**Fig. 2:**
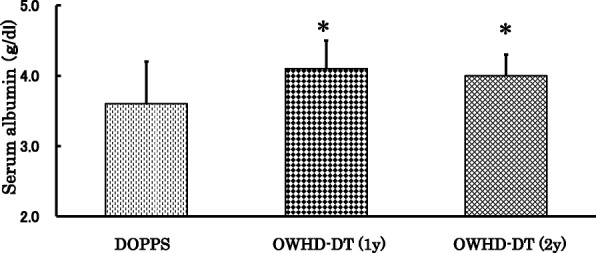
Serum albumin concentrations at 1 year and 2 years in patients on once-weekly hemodialysis and diet therapy (OWHD-DT), compared with International Dialysis Outcomes and Practice Patterns Study (DOPPS) patients. ✻*p* < 0.001

**Fig. 3:**
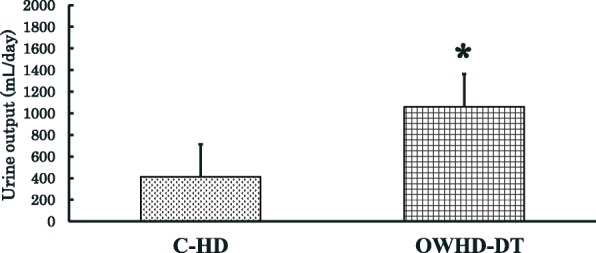
Comparison of residual urine output at 12 months after initiation of hemodialysis between patients on conventional hemodialysis (C-HD) and those on once-weekly hemodialysis and diet therapy (OWHD-DT). ✻*p* < 0.001

Mean serum albumin concentrations in patients on OWHD-DT were 4.1 ± 0.4 g/dL at 12 months and 4.0 ± 0.3 g/dL at 24 months. These values were higher than the value of 3.6 ± 0.6 g/dL of patients enrolled in the international Dialysis Outcomes and Practice Patterns Study (DOPPS) [28], (Fig. 2). The patients on OWHD-DT had significantly greater urine output than those on C-HD at 12 months after the initiation of HD (1161.5 ± 301.6 vs 412.0 ± 299.8 mL/day, *p* < 0.001, Fig. 3), and mean clearance of creatinine and urea was 2.1 ± 0.6 vs 0.7 ± 0.5 ml/minute, *p* < 0.001).

### Time required and cost for medical treatment

**Fig. 4:**
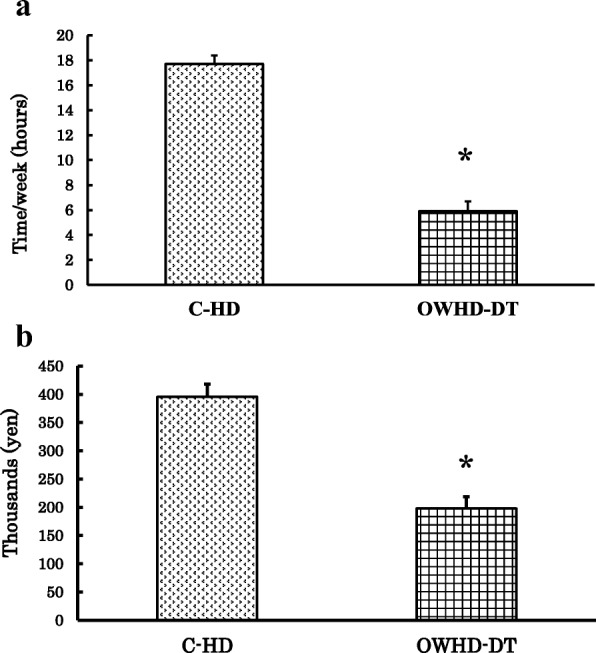
Comparisons of time required for medical treatment including both dialysis and hospital attendance, which means the time per week for dialysis therapy plus the time for round-trip from home to hospital (**a**), and overall monthly medical expenses (**b**) between patients on conventional hemodialysis (C-HD) and those on once-weekly hemodialysis and diet therapy (OWHD-DT). ✻*p* < 0.001

We compared the time required for medical treatment including both dialysis and hospital attendance, which means the time per week for dialysis therapy plus the time for round-trip from home to hospital, between the patients on OWHD-DT and the 30 prevalent patients on C-HD. The average time required for medical treatment was 5.9 ± 0.8 h for the patients on OWHD-DT and 17.7 ± 0.7 h for those on C-HD (*p* < 0.001). As expected from the once-weekly versus thrice-weekly HD, OWHD-DT patients required one third of the time for medical treatment compared with C-HD patients (Fig. 4a).

We also compared the monthly costs of treatment between the patients on OWHD-DT and those on C-HD. The average monthly cost of treatment, including facility services, professional services, and outpatient pharmacy, was 198.1 ± 21.7 thousand yen for those on OWHD-DT and 399.2 ± 22.3 thousand yen on controls on C-HD (*p* < 0.001). The medical expenses for patients undergoing OWHD-DT were about 50% lower than those on C-HD (Fig. 4b).

### Patient survival

**Fig. 5:**
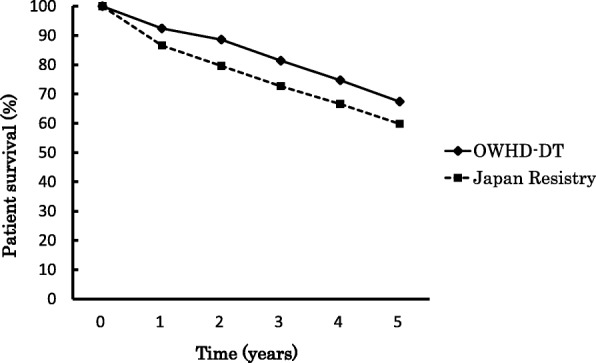
Comparison of patient survival between patients on once-weekly hemodialysis and diet therapy (OWHD-DT) with patients from the Japan Registry. Log rank *p* < 0.001

Extended follow-up analysis of the patients showed survival rates of 92.4, 88.6, 81.4, 74.7, and 67.4% at 1, 2, 3, 4, and 5 years, respectively, including time after transition to twice-weekly or thrice-weekly HD. The survival rates of the patients on OWHD-DT were better than those patients in the Japan Registry which are 86.6, 79.4, 72.5, 66.0, and 59.4% at 1, 2, 3, 4, and 5 years respectively. The Japan Registry contains 36,711 patients who had newly started maintenance hemodialysis in 2007 [29] (Fig. 5).

## Discussion

In this paper, we report the possibility that once-weekly dialysis can be successful if combined with strict low-protein and low-salt DT that can be well adhered to for patients with ESRF, whose renal function has diminished to the point that uremia cannot be controlled by the DT alone. OWHD-DT may be a safe and cost-effectiveness regimen that provides more HD-free time for selected patients. The main reasons for dropping out were loss of dietary adherence and/or reduction of residual renal function. We were unable to show any adverse effects of this treatment strategy, and the clinical outcomes were actually somewhat better than the overall outcomes of incident patients in Japan. The absence of adverse events may have been due to preferential selection of patients who had high motivation to adhere to the strategy and some residual renal function.

In spite of the fact that thrice-weekly HD became the standard method to treat patients with ESRF in the mid-1970s, the efficacy of once-weekly HD in combination with a very low-protein diet supplemented with essential amino acids and ketoacids was reported by Mitch et al. [23], Morelli et al. [24] and Locatelli et al. [25] in the 1980s and 1990s. Those studies reported that infrequent dialysis could have some beneficial points compared to thrice-weekly HD programs begun suddenly during the conservative treatment phase. These benefits include better psychological adaptation to the need for dialysis, better acceptance of conventional HD programs when needed later, and a reduction in costs and organizational problems for both patients and the National Health Service. More recently, Caria et al. reported a “Combined Diet Dialysis Program” consisting of the application of once-weekly dialysis plus a 0.6 g/kg/day low-protein diet. In this study, at the 24-month follow-up, 39.4% of the patients were still on the program and the survival rate was 94.7% [26]. Bolasco et al. also recognized the benefits of beginning maintenance dialysis once weekly with incremental transition to more-frequent dialysis therapy [30].

One of the primary purposes of dialysis therapy is to control elevated serum concentrations of uremic solutes, potassium, phosphate, and the excessively accumulated fluids seen in ESRF patients. The National Cooperative Dialysis Study showed the benefit of maintaining lower serum urea nitrogen concentrations in HD patients [1]. The extents of these abnormalities seen in HD patients depend not only on the dialysis dose but also on the rate of accumulation of these substances during the inter-dialytic interval. Regarding the delivered dose of HD, recent guidelines recommend a target spKt/V of 1.2–1.4 per HD. The hemodialysis (HEMO) study showed that there was no benefit from more intensive HD (higher per-session Kt/Vurea) when patients underwent thrice-weekly HD [3]. The mean spKt/V of the OWHD-DT patients in our study was 1.34 ± 0.13, indicating that the dialysis dose for one session of HD is adequate.

The rate of accumulation of fluids and toxic metabolites may largely depend on the ingested quantities from foods and drinks during the inter-dialytic interval. A reduction in dietary protein intake may not only reduce the generation of deleterious nitrogenous metabolic products but could also lead to a concomitant reduction in the intake of both phosphorus and potassium. While solute removal can be augmented by increasing the frequency of HD sessions, rapid accumulation of toxic metabolites and fluids can be prevented by low-protein and low-salt DT. Therefore, the frequency of HD sessions could be reduced by DT. Thus, diet appears to affect the delicate balance of HD frequency in conflicting ways [31].

Good adherence with the diet is essential for safe and successful OWHD-DT. Previously low-protein diets with sufficient energy intake were demonstrated to have no deleterious effect on nutritional status in patients with chronic renal failure [32]. Therefore, reducing protein intake while keeping energy intake stable by increasing carbohydrates and fats can maintain good nutritional status, and prevent the need for higher doses of dialysis. However, because protein-energy wasting is a life-threatening problem in HD patients [33], more investigations is needed to ensure that reducing protein intake is nutritionally safe in the long-term care of dialysis patients. Davies et al. examined both energy and protein intake and reported that despite protein intake being less than that recommended by Kidney Disease Outcomes Quality Initiative guidelines, nutritional status was well maintained in peritoneal dialysis patients whose energy intake was sufficient [34]. In our study, the patients on OWHD-DT maintained the same levels of BMI, serum albumin and serum transferrin throughout of the treatment course. Moreover, the serum albumin levels at 1 and 2 years in the patients on OWHD-DT were actually significantly higher compared with those of the DOPPS patients. Hence, the nutritional status of the patients on OWHD-DT could not be considered to have deteriorated despite low-protein diets. Many protein-reduced products that are made up of carbohydrates and are substantially free of nitrogen compared with regular foods have become commercially available and more palatable [35, 36]. The study patients actively incorporated these food products into their diets. Therefore, even under restricted protein intake, sufficient energy intake contributes to the maintenance of good nutritional status.

Residual renal function in HD patients has been demonstrated to be a crucial predictor of clinical outcomes, and higher residual urine output has been reported to be significantly associated with a lower risk of death in dialysis patients [37–39]. Furthermore, it has been reported that patients starting maintenance HD therapy two times per week was associated with a slower decline of residual renal function compared with those starting HD three times per week [40, 41]. Given this background, infrequent dialysis has been proposed as a new paradigm for HD initiation [42]. In this present study, the patients on OWHD-DT had significantly higher urine output than those on C-HD at 1 year after starting HD. This fact is consistent with previous reports stating that less-frequent HD was beneficial for preserving residual renal function. However, it is unclear whether OWHD-DT can preserve residual renal function significantly longer than twice-weekly HD. In addition, arteriovenous fistula (AVF) creation in ESRF patients have been shown to be associated with a slowing the decline of the estimated glomerular filtration rate, and it has been suggested that the plausibility of the ‘downstream’ effects by which the fistula may reach renal vascular beds at both the macro- and microvascular levels [43]. In this study, all patients had functional AVF, and it may contribute to preserve their residual renal function.

The prevalence and incidence of end-stage renal disease are increasing, and renal replacement therapy consumes a significant proportion of health care resources in all over the world [44, 45]. This study showed a decrease in medical expense of about 50% on OWHD-DT than on C-HD. Consistent with our data, Caria et al. also reported that CDDP, namely once-weekly HD combined with a low-protein diet, resulted in an approximate 50% savings on the cost of medications and dialysis [26]. Hence, OWHD-DT may also be beneficial from an economic point of view.

In our study, the survival rates of the patients on OWHD-DT were better than those of the Japan Registry for the patients who had just started maintenance HD in 2007. Control of body fluid volume must be primarily important issue to achieve adequate maintenance of dialysis patients. To deal with it, Kt/V concept is meaningless, and salt restriction should be incorporated [46] In this study, the patients who excellently adhered to low salt intake could survive on OWHD-DT protocol for a long time, showing their interdialytic weight gain around 2.0 kg over the course of the entire week between treatments. While there is greater medical risk and increased mortality in undernourished dialysis patients, particularly those with hypoalbuminemia, the patients in our study on OWHD-DT had acceptable levels of serum albumin even compared with the DOPPS patients. Furthermore, preserved renal function is suggested to be associated with improved survival among HD patients and the patients on OWHD-DT had greater urine output than those of C-HD patients. Moreover, serial measurements of hemoglobin, urea nitrogen, potassium, phosphate, intact parathyroid hormone, β^2^-microglobulin, inter-dialytic body weight gain, blood pressure and cardiothoracic ratio were also maintained in acceptable ranges in the studied patients. Furthermore, the levels of serum CRP in the studied patients were quite low, considering they were hemodialysis patients. All these factors likely contribute to the favorable survival rates of the studied patients. The studied patients all were originally selected patients who had motivation to control their diet, and they were repeatedly received nutritional counselling by a renal dietician once or twice a month. Moreover, physicians gave more careful observations to them than to general population on C-HD. An “enrollment effect” like this may contribute to the better survival of OWHD-DT patients.

One limitation of this OWHD-DT program is that it is not appropriate for every ESRF patient, but only for selected patients who are highly motivated to choose the therapeutic modality and are able to strictly adhere to DT. Moreover, the low-protein diet is safe only in the hands of a multidisciplinary and dedicated staff who thoroughly understand the therapy, including specialized renal dietitians who also have constant oversight. From this point of view, this treatment is likely only to be feasible at a relatively limited number of facilities.

## Conclusions

OWHD-DT may be a safe and cost-effective regimen that provides more HD-free time for selected patients with ESRF. In addition to favorable clinical and economic outcomes, an incremental transition to twice- or thrice- weekly HD may create a less stressful transition for the patient to dialysis therapy. However, it must be emphasized that the benefit of OWHD-DT was limited, since only a subset of patients (24%) could be maintained on this treatment after 2 years.

This treatment cannot be seen as a general maintenance strategy for patients with ESRF, but may represent a favorable option for use with carefully selected, highly motivated patients, with access to continuous support from trained medical staff, especially nutritionists who are experts in prescribing and assisting the maintenance of low-protein, low-salt diets that also provide adequate energy intake.

## Abbreviations

AVF: Arteriovenous fistula
BMI: Body mass index
C-HD: Conventional thrice-weekly hemodialysis
CRP: C-reactive protein
CTR: Cardiothoracic ratio
DT: Dietary treatment
HD: Hemodialysis
IDW: Interdialytic weight gain
OWHD-DT: Once-weekly hemodialysis combined with dietary treatment
spK/TV: Single pool K/TV
